# Modulation of Metastasis and Stemness-Associated Genes by Heme Oxygenase 1 (HO-1) in Prostate Cancer

**DOI:** 10.1200/GO.22.36000

**Published:** 2022-05-05

**Authors:** Agustina Sabater, Ayelen Toro, Pablo Sanchis, Gaston Pascual, Rocio Seniuk, Elba Vazquez, Javier Cotignola, Geraldine Gueron, Juan Bizzotto, Sofia Lage-Vickers

**Affiliations:** ^1^ University of Buenos Aires, Buenos Aires, Argentina

## Abstract

Prostate cancer stem cells enhance the tumor's capacity to proliferate, metastasize, and to resist therapies. Thus, stemness represents a therapeutic challenge in prostate cancer (PCa). We have previously reported a strong anti-tumoral effect of heme oxygenase 1 (HO-1) in prostate carcinogenesis in vivo and in vitro. However, its association with metastasis-stemness is still poorly elucidated. Thus, we aimed at describing the effects of HO-1 overexpression on metastasis/stemness processes in PCa, by also identifying key HO-1-modulated genes.

**METHODS:**

We performed clonogenic assays to evaluate the effect of HO-1 pharmacological induction on the stem properties of PC3-cells. We carried out an RNA-seq analysis to assess the transcriptional differences of 144 metastasis/stemness/STAT3-signature genes after HO-1 induction. The clinical significance of HO-1-modulated genes was assessed by comprehensive bioinformatics analyses using public data repositories with gene expression and survival data of PCa patients (Oncomine, n = 1,128; TCGA-PRAD, n = 565). RNA-seq results were validated by RT-qPCR.

**RESULTS:**

Clonogenic assays results showed a significant reduction in colony formation efficiency after HO-1 pharmacological induction with hemin (*P* ≤ .01). Transcriptomics profiling by RNA-seq evidenced a significant modulation of 32 key markers related to metastasis/stemness/STAT3-signature under HO-1 induction. Further, 15 HO-1-modulated genes were differentially expressed in PCa vs. normal prostate gland (Oncomine) and HO-1 induction reverted the expression profile observed for these genes. We next used the TCGA-PRAD dataset which revealed that ADAM15, BCL2L1, LTBR, MBNL2 and SPINT1 had the same expression profile as in Oncomine. Additionally, PCa patients with high MBNL2 expression showed a longer disease progression-free survival, which is interesting as it is upregulated in PC3-cells overexpressing HO-1, as validated by RT-qPCR.

**CONCLUSION:**

Here, we propose a potential mechanism by which HO-1 could inhibit metastasis/stemness by modulating relevant genes in PCa progression. This study may cast a new light on PCa treatment, highlighting HO-1 modulation of cell plasticity, supporting it as a potential therapeutic target for the disease.

